# Feasibility of a web-based neurocognitive battery for assessing cognitive function in critical illness survivors

**DOI:** 10.1371/journal.pone.0215203

**Published:** 2019-04-12

**Authors:** Kimia Honarmand, Sabhyata Malik, Conor Wild, Laura E. Gonzalez-Lara, Christopher W. McIntyre, Adrian M. Owen, Marat Slessarev

**Affiliations:** 1 Department of Medicine, Western University, London, Ontario, Canada; 2 Faculty of Science, Western University, London, Ontario, Canada; 3 Brain and Mind Institute, Western University, London, Ontario, Canada; 4 Department of Medical Biophysics, Western University, London, Ontario, Canada; University of Pittsburgh, UNITED STATES

## Abstract

**Purpose:**

To assess the feasibility of using a widely validated, web-based neurocognitive test battery (Cambridge Brain Sciences, CBS) in a cohort of critical illness survivors.

**Methods:**

We conducted a prospective observational study in two intensive care units (ICUs) at two tertiary care hospitals. Twenty non-delirious ICU patients who were mechanically ventilated for a minimum of 24 hours underwent cognitive testing using the CBS battery. The CBS consists of 12 cognitive tests that assess a broad range of cognitive abilities that can be categorized into three cognitive domains: reasoning skills, short-term memory, and verbal processing. Patients underwent cognitive assessment while still in the ICU (n = 13) or shortly after discharge to ward (n = 7). Cognitive impairment on each test was defined as a raw score that was 1.5 or more standard deviations below age- and sex-matched norms from healthy controls.

**Results:**

We found that all patients were impaired on at least two tests and 18 patients were impaired on at least three tests. ICU patients had poorer performance on all three cognitive domains relative to healthy controls. We identified testing related fatigue due to battery length as a feasibility issue of the CBS test battery.

**Conclusions:**

Use of a web-based patient-administered cognitive test battery is feasible and can be used in large-scale studies to identify domain-specific cognitive impairment in critical illness survivors and the temporal course of recovery over time.

## Introduction

Long-term cognitive impairment is a common complication in critical illness survivors [1–3] but its natural history and the temporal profile of cognitive recovery in individual patients remain unknown. Understanding these features is critical for identifying optimal therapeutic windows and selecting patient subgroups most likely to benefit from targeted interventions.

Several studies have examined cognitive function in the ICU patients at various time points ranging from assessments in hospital at time of or shortly after ICU discharge [4–8] to long-term follow-up of up to 13 years after discharge [9]. Limitations of existing neurocognitive assessment tools have restricted cognitive testing in prior studies to a few discrete time points [1–9]. Many neurocognitive tools require specially trained staff for test administration, take a long time to administer, and necessitate patients to attend testing sessions in person, which often excludes those who have limited mobility, are institutionalized, live far away from the testing centre, or are unwilling to return to the hospital where they were admitted due to associated traumatic memories. Attrition rates for studies involving long-term follow-up of ICU patients vary from 60 to 79% at 3 months and 69 to 95% at 12 months [1, 6, 10–13]. High attrition rates in some studies that require ICU patients to return to clinic for follow-up testing may affect the generalizability of study findings. A web-based neurocognitive assessment tool that can be self-administered remotely by patients may lead to higher follow-up rates and enable large-scale natural history studies of long-term cognitive impairment in critical illness survivors.

We assessed the feasibility of using a widely validated, web-based neurocognitive test battery previously used in large cohort studies, Cambridge Brain Sciences (CBS) [14–16], as well as in other clinical populations [17–22], in a cohort of critical illness survivors.

## Materials and methods

### Study design and setting

We conducted a prospective observational study of patients admitted to two adult tertiary care centre intensive care units (ICUs) in London, Canada. The study was approved by our local research ethics board (WesternREM, study identification number 108156).

### Patients

We included adult patients (18 to 80 years of age) who were mechanically ventilated for a minimum of 24 hours. The latter criterion excluded routine post-operative patients and non-critically ill patients requiring a brief period of mechanical ventilation (e.g. for acute intoxication).

Patients were excluded if they had a pre-existing diagnosis of dementia, new or pre-existing diagnosis of neurological disease known to affect cognitive function (e.g. stroke, head trauma, intracranial hemorrhage, traumatic brain injury, or intracranial malignancy), impaired vision or significant upper extremity weakness precluding use of a computer, inability to communicate in English or were unable to or declined to provide informed consent, or active delirium as determined by the Intensive Care Delirium Screening Checklist (ICDSC) [23].

### Measurements

#### Cognitive assessment

CBS is a web-based neurocognitive assessment battery consisting of 12 tests that tap into a broad range of cognitive processes. These include: Feature Match, Odd One Out, Polygons, Rotations, Spatial Planning, Monkey Ladder, Paired Associates, Spatial Span, Spatial Search, Digit Span, Double Trouble, and Grammatical reasoning. Each of the 12 tests taps into three cognitive domains–reasoning skills, short-term memory, and verbal ability—to varying degrees. Previously, it was shown that these three factors–reasoning skills, short-term memory, and verbal ability–explain a large proportion of variability in performance across the tests [15]. The CBS can be self-administered by individuals without the need for a neuropsychologist or other trained personnel. The battery has been widely used as a measure of cognitive function in individuals across the world [14–16], as well as those with neuroanatomical lesions [17–18] and Parkinson’s disease [19–20].

#### Informant Questionnaire on Cognitive Decline in Elderly

To screen for previous history of cognitive deficits, we asked a relative or friend who knows the patient well to complete the Informant Questionnaire on Cognitive Decline in Elderly (IQCODE). The IQCODE has been shown to have high reliability in measuring cognitive decline and correlates well with other neurocognitive tests [24]. Family assessors are presented with 16 specific tasks (e.g., remembering where things are usually kept) and asked to rate the patient’s current status on that task relative to 10 years ago on a scale of 1 (much improved) to 5 (much worst) with a score of 3 indicating “no change”. A cut-off score of 3.44 was used as a positive screen for pre-existing dementia [9].

#### Feasibility

Our aim was to assess the feasibility of using a web-based, neurocognitive battery to assess cognition in ICU survivors at the time of ICU discharge. Our primary measure of feasibility was the number of patients who completed the entire 12-test cognitive battery. In addition, researchers who recruited patients and were present while patients underwent cognitive testing were asked to make notes about any issues that arose throughout the administration of the cognitive battery and any concerns that were raised by patients throughout testing so that these challenges could be addressed in future studies using the CBS battery.

### Study protocol

One investigator (KH or SM) or a research assistant screened patients for eligibility using medical charts and discussion with bedside nurses. We approached eligible patients and obtained written informed consent prior to commencing the study. Patients were deemed to be adequately alert and capable of providing consent if they were able to participate in the consent discussion.

We recorded demographic and clinical variables including age, sex, admission diagnosis, hospital and ICU admission dates, and Nine Equivalents of Nursing Manpower Use Score (NEMS) [25] on the day of testing from each patient’s chart or electronic medical record.

Patients completed cognitive assessment using a laptop computer with an attached computer mouse in their hospital bed in the ICU or shortly after discharge to the ward. Investigators assisted patients by creating a personalized login and password on the CBS study webpage. A standardized set of written and pictorial instructions and a short instructional video preceded each cognitive test. Patients were provided with as much time as they needed to review the instructions prior to beginning each test. Patients completed each of the tests in sequence until the entire battery of 12 tests was completed or they were unable to continue due to testing related fatigue.

We asked a relative or friend who knew the patient well to complete the IQCODE. Family members responded to the IQCODE questions by telephone if they were not available in person.

### Data analysis

Demographic and clinical variables were reported using descriptive statistics, expressed as frequency and percentage for categorical variables and mean and standard deviation (SD) or median and interquartile range (IQR) for continuous variables as applicable.

Patients were defined as having cognitive impairment on a given test if their raw score was ≥ 1.5 SDs below age- and sex-matched controls derived from the CBS normative database [15]. We then compared patients’ cognitive performance with available data from healthy age- and sex-matched control data by converting raw scores into z-scores.

To determine patients’ scores on each of the three cognitive domains (reasoning skills, short-term memory, and verbal processing), the z-score for each individual test was multiplied by a value that reflected the contribution of that test to each cognitive domain (i.e., factor loading) as established by Hampshire and colleagues [15]. Patients’ overall score on each cognitive domain was therefore the sum of the weighted (factor loaded) scores for that domain across all 12 tests. Data imputation was only used to compare domain scores for patients who completed all 12 tests. The scores are designed such that the healthy population mean on each cognitive domain is 0 and the SD is 1.0.

Finally, we defined an abbreviated CBS battery that included six of the 12 CBS tests which most strongly reflect one of the three cognitive domains based on previously published data (Reasoning Skills: Odd One Out and Rotations; Short-term Memory: Paired Associates and Monkey Ladder; and Verbal Processing: Digit Span and Verbal Reasoning) [15]. We replaced the scores for the omitted tests with their expected values given the six observed test scores and the known correlation structure among the tests in the population. The correlation structure between the 12 tests in the CBS battery was derived from a sample of 44,600 [15]. This method has been shown to be most accurate when calculating principle component analysis scores in the presence of missing data [26]. We then calculated a z-score for each patient on each of the three cognitive domains based on the abbreviated CBS and compared this score to the z-scores calculated based on the complete CBS battery.

## Results

**Fig 1:**
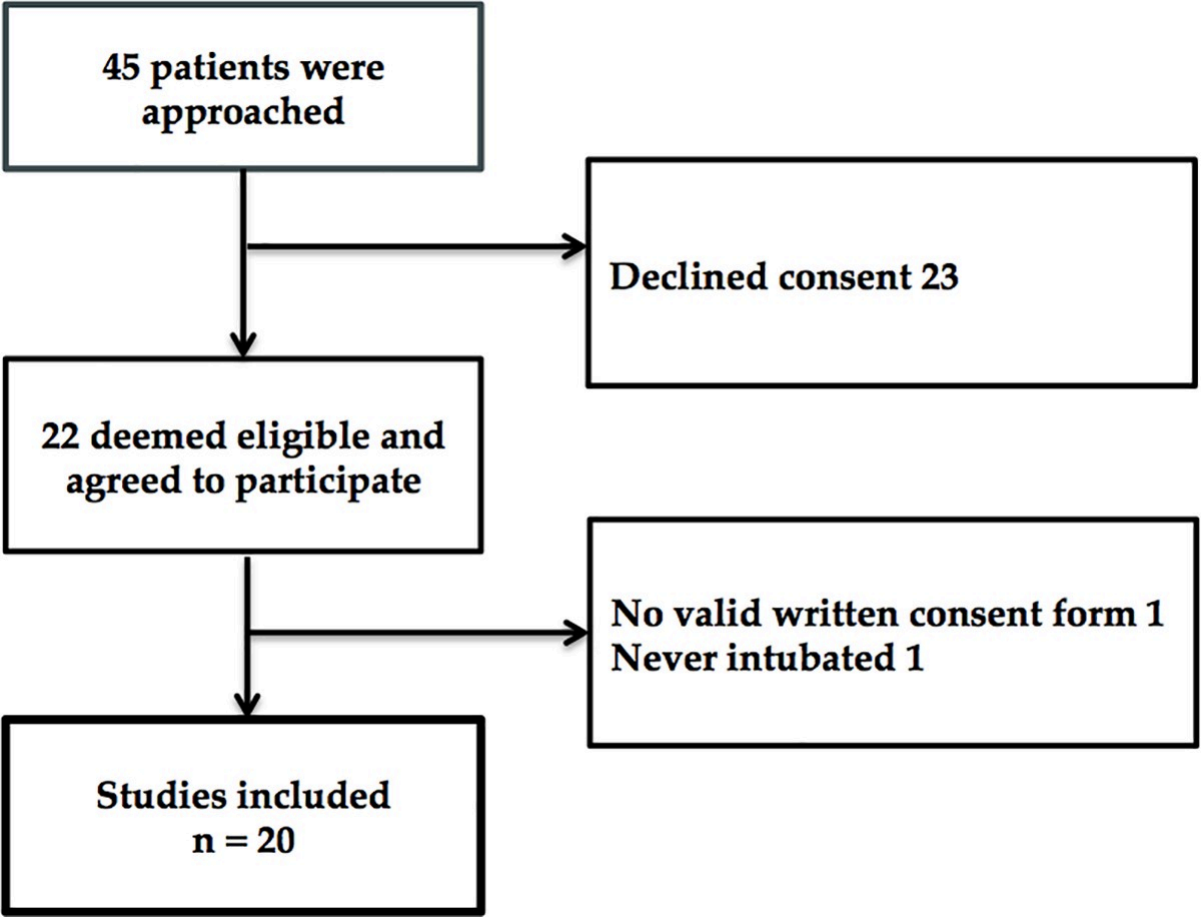
Recruitment flow diagram.

Of 45 patients approached, 25 declined to participate in this study, most commonly due to self-reported inability to participate or limited availability due to required clinical care activities (e.g., diagnostic tests). Twenty patients (7 females) were included in the analysis (Fig 1). Patient demographic and clinical characteristics are presented in Table 1. We reached family members or friends of 15 patients for completion of the IQCODE. None of these patients were found to have pre-morbid dementia as assessed by the IQCODE.

**Table 1:** Patient demographic and clinical characteristics.

CHARACTERISTIC		STATISTIC
Age, median (IQR)		58.5 (41–66)
Sex		
	Males	13
	Females	7
Admission Diagnosis		
	Respiratory, *n*	3
	Cardiac, *n*	2
	Sepsis, *n*	2
	Cardiac arrest, *n*	4
	Neurologic, *n*	1
	Trauma, *n*	2
	Surgical, *n*	3
	Other, *n*	3
Duration of Mechanical Ventilation, median (IQR)		2.5 (1–3)
ICU length of stay (d), median (IQR)		5 (4–9)
Nine equivalents of nursing manpower use score, median (IQR)		18 (18–22)

IQR = Interquartile range.

Thirteen patients were tested in the ICU and seven were tested within one to four days of transfer to the ward. Patients were tested after a median of 4 days (IQR 3.75) following ICU admission. Three patients completed testing on two separate days at the patient’s request. Seventeen of 20 patients completed the full 12-test CBS battery (the other three patients completed four, six and seven tests). Of the three patients who did not complete the full battery, two were tested on the ward and one was tested while still in the ICU. The mean duration for completion of the 12-test CBS battery was 45.5 minutes (SD 11.1) excluding the three patients who did not complete the entire battery and one patient whose testing who had a long break between tests.

All patients were impaired on at least two tests, and 18 were impaired on at least three tests relative to healthy controls. Among the 17 of 20 patients who completed the full CBS battery, patients were impaired on a median of eight tests (IQR 4.5–10, range 2–11).

**Fig 2:**
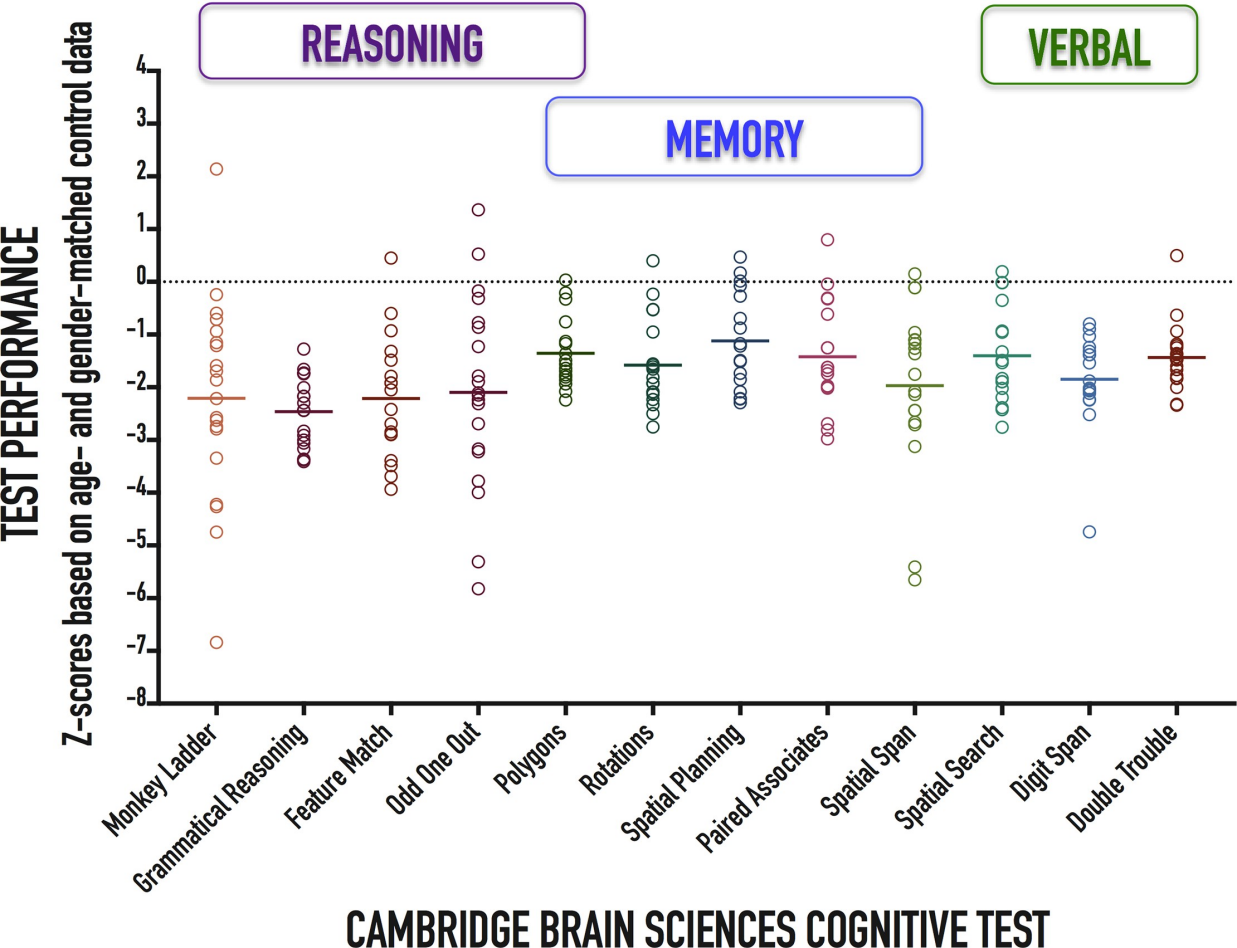
Patient performance on the 12-test Cambridge Brain Sciences neurocognitive battery. Individual patient (circles) and cohort (solid lines) test performance presented as z-scores corrected for age and sex.

**Fig 3:**
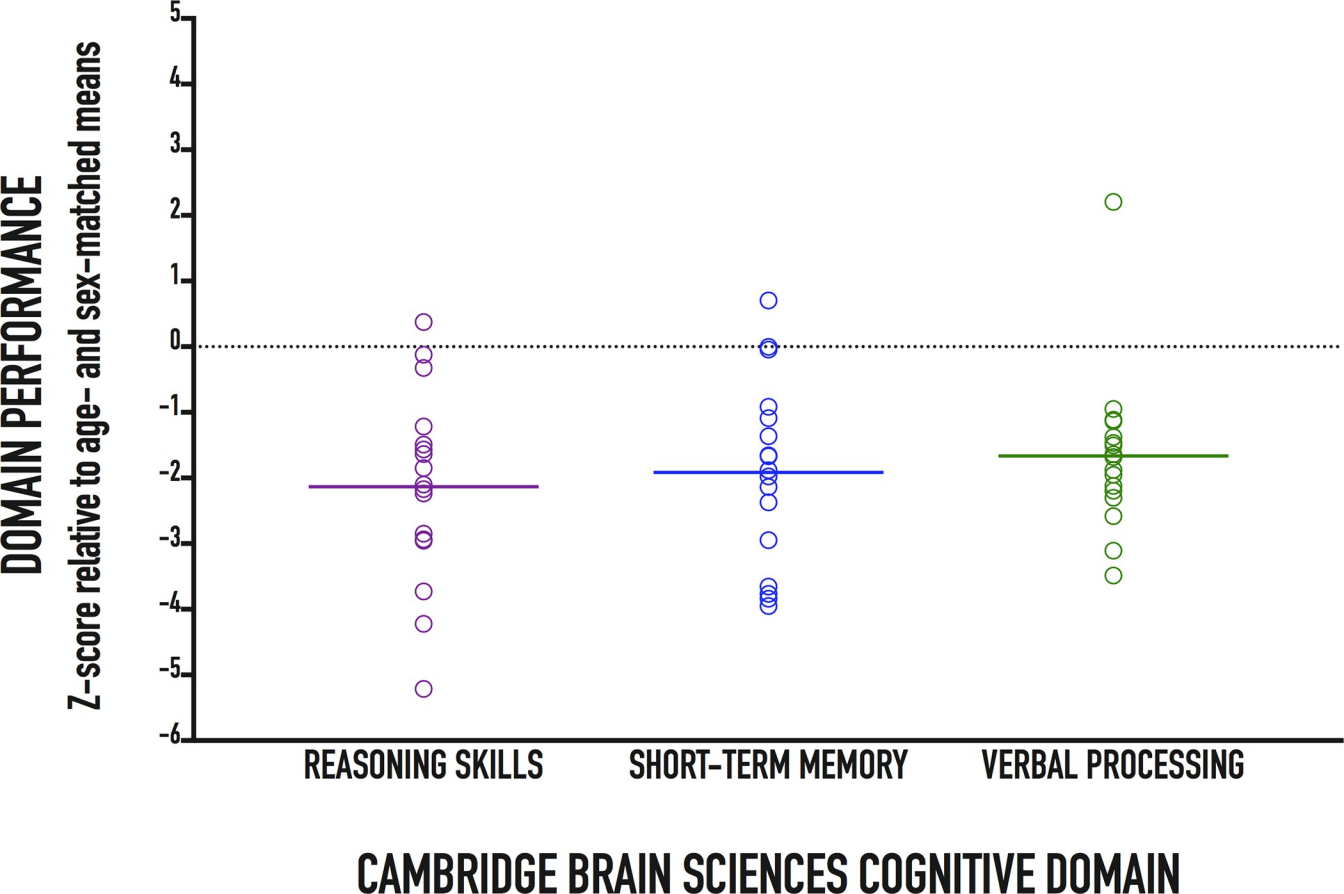
Patient performance on the three Cambridge Brain Sciences cognitive domains: reasoning skills, short-term memory, and verbal processing. Individual patient (circles) and cohort (solid lines) test performance on each cognitive domain presented as z-scores corrected for age and sex. By design, healthy norm mean = 0 and standard deviation = 1.

Individual patient performances as well as cohort means on each of the 12 CBS tests relative to normative data are presented in Fig 2. Patients had poorer performance on all CBS tests compared to healthy controls (Table 2). Patients also had poorer performance on all three cognitive domains compared to healthy controls (Fig 3 and Table 2). Our anonymized data set is provided in S1 Table.

**Table 2:** Cognitive performance of critical care survivors relative to healthy control population.

COGNITIVE TEST/ DOMAIN	MEAN Z-SCORE (SD)	STATISTICS
**CBS COGNITIVE TEST**		
Feature Match	-2.21 (1.16)	t(17) = -8.10, p < 0.0001
Odd One Out	-2.10 (1.83)	t(19) = -5.14, p < 0.0001
Polygons	-1.35 (0.67)	t(16) = -8.28, p < 0.0001
Rotations	-1.58 (0.84)	t(18) = -8.22, p < 0.0001
Spatial Planning	-1.12 (0.92)	t(16) = -5.04, p < 0.0001
Monkey Ladder	-2.21 (1.93)	t(19) = -5.13, p < 0.0001
Paired Associates	-1.42 (1.04)	t(16) = -5.64, p < 0.0001
Spatial Span	-1.97 (1.56)	t(18) = -5.51, p < 0.0001
Spatial Search	-1.40 (0.92)	t(16) = -6.30, p < 0.0001
Digit Span	-1.85 (0.91)	t(16) = -8.40, p < 0.0001
Double Trouble	-1.43 (0.62)	t(19) = -10.40, p < 0.0001
Grammatical Reasoning	-2.46 (0.65)	t(19) = -16.89, p < 0.0001
**COGNITIVE DOMAIN**		
Reasoning Skills	-2.13 (1.45)	t(16) = -6.05, p < 0.0001;
Short-term Memory	-1.92 (1.41)	t(16) = -5.60, p < 0.0001
Verbal Processing	-1.66 (1.21)	t(16) = -5.65, p < 0.0001

We identified two feasibility issues with the web-based CBS platform: First, patient-reported testing related fatigue due to battery length (with three of 20 patients requesting that the testing be stopped before completion of all 12 tests); and second, one patient reported inexperience with use of a computer mouse and was instructed on its use by the investigator.

**Fig 4:**
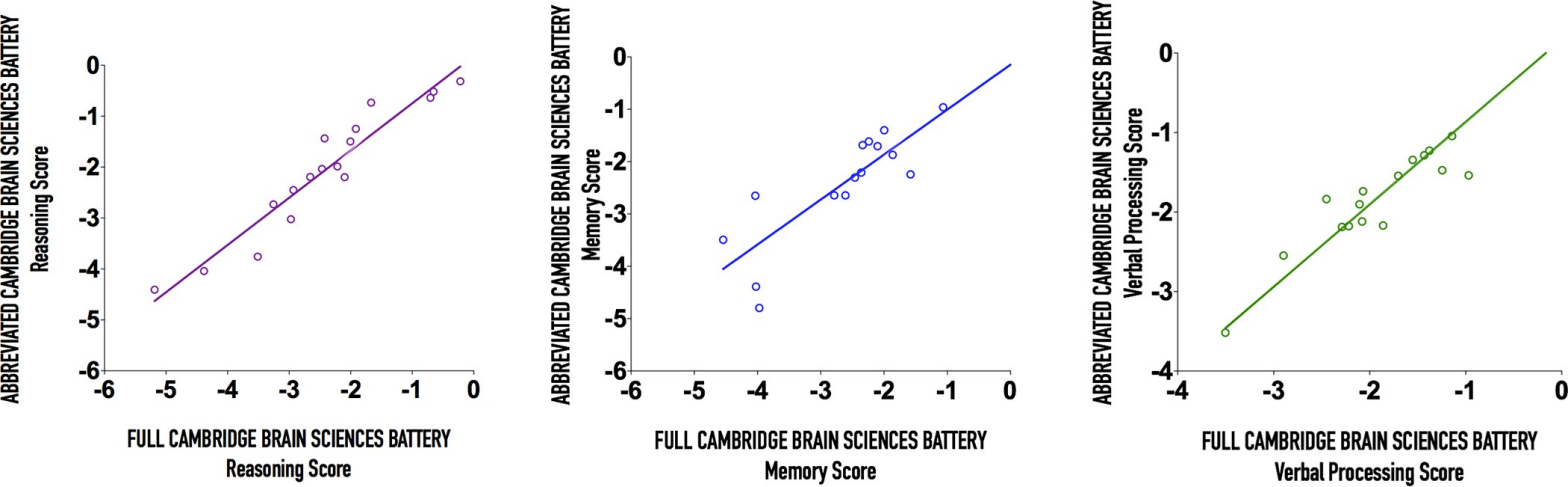
Scatterplots including a linear fit of the abbreviated Cambridge Brain Sciences Battery against the full battery on the three CBS cognitive domains. (A) Reasoning (r = 0.91), (B) Memory (r = 0.80), and (C) Verbal Processing (r = 0.94). Solid black line indicates x = y.

To address the issue of test fatigue related to battery length, we compared the performance of an abbreviated version of the CBS battery containing 6 tests with the full 12-test battery and found that the abbreviated battery performed well in predicting performance on each of the three cognitive domains (Fig 4).

### Discussion

Our results demonstrate that a web-based patient-administered neurocognitive test battery can identify domain-specific cognitive impairment in critical illness survivors. Unlike paper-based cognitive assessment tools, the web-based battery can be self-administered by patients from any location (ward, rehabilitation facility, home) after minimal instructions on logging in and starting the tests, obviating the need for patients to travel to hospital to be tested by specially trained staff. The self-administering nature of web-based battery may enable large-scale studies similar to those completed in other populations [14–16], and provides opportunity for repeated remote monitoring of cognitive recovery in patients who are unable to return to clinic. Given high prevalence of functional disability, post-traumatic stress disorder, and challenging travel logistics, remote web-based testing of cognition can provide a more patient-centred approach to monitoring cognition in ICU survivors.

The majority of previous studies have used traditional, paper-and-pencil testing to assess cognitive function in critical illness survivors [1–9]. Screening tools such as the Mini-Mental State Examination (MMSE) [27], have been designed to screen for overt dementia, but lack comprehensiveness and have poor sensitivity for more subtle, yet consequential cognitive deficits in critical illness survivors [28].

More comprehensive cognitive batteries such as the Repeatable Battery for the Assessment of Neuropsychological Status (RBANS) [29] and Wechsler Adult Intelligence Scale-Fourth Edition (WAIS-IV) [30], have well-established psychometric properties (RBANS [31,32]; WAIS-IV [33,34]) have been widely used in various patient populations (RBANS [31,32,35,36]; WAIS-IV [37,38]), but are not optimal for some natural history studies of critical illness survivors due to the need for in-person testing by trained personnel. Our results demonstrate that web-based cognitive testing is both feasible and able to detect subtle changes in cognitive domains similar to traditional paper-and-pencil tests.

There are several advantages to the CBS battery that make it an attractive tool for assessing cognitive dysfunction in ICU survivors. Similar to traditional cognitive assessment tools, the CBS battery used in this study is comprehensive, with 12 individual tests assessing cognitive function across three distinct cognitive domains. It has been used to study cognition in several large patient cohorts [14–16] and has an established large normative database to enable sex- and age-matched comparisons of patients’ performance to healthy controls. Furthermore, the CBS battery can be administered in the functional magnetic resonance imaging scanner, enabling mapping of cognitive results to neuroimaging data [14,39].

Optimization of the CBS battery for ICU needs by addressing feasibility issues identified in this study would enable its use for remote monitoring of cognitive recovery in individual patients, and provide an objective outcome metric for tracking patient’s cognitive health and gauging effectiveness of candidate preventative, therapeutic, and rehabilitative interventions. Given that repeated testing can lead to practice effects, the CBS battery has been optimized for repeated measures studies by incorporating proprietary algorithms that generate a unique set of problems for each assessment, facilitating longitudinal studies with minimal ‘practise effects’. However, whether repeated testing leads to practice effects in ICU survivors requires further dedicated studies. Furthermore, given comparable performance of an abbreviated 6-test version of the CBS battery in our study, its use in future studies would address the issue of test fatigue related to battery length. While we did not administer the abbreviated 6-test battery in this study, we expect that its duration will be approximately half of the 12-test battery (i.e., approximately 20 minutes). This will need to be confirmed in dedicated future studies. In addition, the CBS has recently become available for use on a portable tablet computer, which may further simplify its administration to ICU patients by obviating the need for the hand-computer mouse coordination.

This study has several limitations. Our small sample size allowed us to determine the feasibility of web-based testing using the CBS battery, but prevents us from drawing any definitive conclusions regarding cognitive outcomes in patients. In the future, we will use our feasibility data to optimize CBS battery for ICU use and repeat cognitive assessment in a larger ICU cohort, with repeated measurements in the same patients to assess the ability to track the cognitive recovery process over time.

Web-based cognitive testing in general is not without limitations. The inability to monitor patients during testing to determine their level of engagement, patients’ access to and inexperience with use of a computer, and attrition in follow-up studies are limitations inherent to the use of web-based (remote) cognitive testing that must be addressed prior to large-scale use of such testing platforms in ICU survivors.

In our study, patients’ performance in our study may have been affected by their ability to use a computer mouse to navigate through the tasks. In future studies, we plan to test tablets as an alternative to computer-mouse combination based on patient preference. Finally, in the absence of a “gold standard” test for cognitive impairment in this study, the psychometric characteristics of the CBS could not be assessed, although previous studies in healthy controls [15], and elderly neuropsychiatric patients [40], have confirmed that it is comparable to standard neuropsychological test batteries in terms of its latent structure and relation to age.

We identified that the length of the CBS cognitive battery may be one challenge to its feasibility in larger studies. The proposed abbreviated CBS battery is one potential solution to this challenge. Future studies are needed to assess the feasibility of the abbreviated CBS battery.

## Conclusions

We demonstrated that a comprehensive, web-based neurocognitive testing platform is feasible for use in critical illness survivors and detects domain-specific cognitive impairment. We identified battery length as a potential challenge to wider scale use of the CBS battery, which we believe can be addressed by shortening the battery length. Further studies using the CBS cognitive battery are needed to determine its feasibility in assessing cognition and cognitive recovery over time. Further optimization of this tool for ICU patients may possibly establish a novel frontier in ICU cognition research by enabling remote monitoring of cognitive recovery in critical illness survivors, correlation of cognitive scores with neuroimaging data to help identify underlying neural mechanisms, and objective assessment of outcomes associated with preventative, therapeutic, and rehabilitative interventions.

## Supporting information

S1 Table Anonymized data set. (XLSX)
